# The effects of selective serotonin reuptake inhibitors on brain functional networks during goal-directed planning in obsessive–compulsive disorder

**DOI:** 10.1038/s41598-020-77814-4

**Published:** 2020-11-26

**Authors:** Minah Kim, Wi Hoon Jung, Geumsook Shim, Jun Soo Kwon

**Affiliations:** 1 Department of Neuropsychiatry, Seoul National University Hospital, Seoul, Republic of Korea; 2 Department of Psychiatry, Seoul National University College of Medicine, 101 Daehak-no, Chongno-gu, Seoul, 03080 Republic of Korea; 3 Department of Psychology, Daegu University, Gyeongsan, Republic of Korea; 4 KAIST Clinic Pappalardo Center, KAIST, Daejeon, Republic of Korea; 5 Institute of Human Behavioral Medicine, SNU-MRC, Seoul, Republic of Korea

**Keywords:** Neuroscience, Psychology, Biomarkers

## Abstract

Whether brain network connectivity during goal-directed planning in patients with obsessive–compulsive disorder (OCD) is abnormal and restored by treatment with selective serotonin reuptake inhibitors (SSRIs) remains unknown. This study investigated whether the disrupted network connectivity during the Tower of London (ToL) planning task in medication-free OCD patients could be restored by SSRI treatment. Seventeen medication-free OCD patients and 21 matched healthy controls (HCs) underwent functional magnetic resonance imaging (fMRI) while performing the ToL task at baseline and again after 16 weeks of SSRI treatment. Internetwork connectivity was compared across the groups and treatment statuses (pretreatment versus posttreatment). At baseline, compared with the HCs, the OCD patients showed lower internetwork connectivity between the dorsal attention network and the default-mode network during the ToL planning task. After 16 weeks of SSRI treatment, the OCD patients showed improved clinical symptoms accompanied by normalized network connectivity, although their improved behavioral performance in the ToL task did not reach that of the HCs. Our findings support the conceptualization of OCD as a network disease characterized by an imbalance between brain networks during goal-directed planning and suggest that internetwork connectivity may serve as an early biomarker of the effects of SSRIs on goal-directed planning.

## Introduction

Recurrent intrusive thoughts and repetitive behaviors are characteristic symptoms of obsessive–compulsive disorder (OCD), for which a neurobiological basis of aberrant frontal-subcortical circuits and related cognitive dysfunctions have been widely reported^1–3^. A consistently reported cognitive dysfunction in OCD patients is goal-directed planning, which refers to the ability to organize intermediate steps to achieve a goal^4,5^, and impaired goal-directed planning abilities affects quality of life and daily functioning as well as treatment response^6,7^. However, whether impaired goal-directed planning is a trait marker or a treatment-modifiable characteristic of OCD remains controversial. While several studies have reported impaired planning capacities in unaffected first-degree relatives of OCD patients^8,9^, other studies have not reported such findings^10,11^. Longitudinal studies investigating whether planning capacity could be restored by cognitive-behavioral therapy (CBT) in child and adolescent OCD patients have yielded favorable results^12–14^. Only one study, however, tested the effects of selective serotonin reuptake inhibitors (SSRIs), the first-line treatment for adult OCD patients, and did not find significant changes in planning capacity over time^15^.

These inconsistencies may result from the generality of the behavioral phenotype despite the complex mechanism of goal-directed planning and pathophysiology of OCD. Thus, brain biomarker techniques such as neuroimaging, which measures brain activation during cognitive functioning, and more direct reflections of pathophysiology than the behavioral phenotype are gaining attention for their ability to detect early brain changes related to the treatment of psychiatric disorders^16,17^. In this context, our previous longitudinal resting-state functional magnetic resonance imaging (fMRI) study demonstrated that in medication-free OCD patients, the decreased small-world efficiency observed at baseline was normalized after 16 weeks of SSRI treatment^18^. With regard to goal-directed planning, previous studies using the Tower of London (ToL) planning task revealed that impaired planning in OCD patients was associated with reduced responsiveness of the dorsolateral prefrontal cortex (DLPFC), caudate, inferior parietal cortex (IPC), and precuneus, which are brain regions comprising the corticostriatal circuit^19–21^. It was reported that reduced responsiveness was normalized after CBT in pediatric OCD patients^12^, but no study to date has shown the effect of SSRI treatment on brain activity during the ToL planning task in adult patients with OCD.

Because human brain function is composed of interacting neural networks, there is growing interest in identifying large-scale functional brain networks (i.e., internetwork connectivity between brain networks), instead of investigating specific local brain regions linked to a psychiatric disorder^18,22,23^. In patients with OCD, a meta-analysis of resting-state functional connectivity studies reported dysconnectivity among the default-mode network (DMN), frontoparietal network (FPN), and salience network, which are thought to be critical for switching between internal obsessive thoughts and goal-directed behaviors^24^. By expanding the scope of the investigation to changes in internetwork connectivity during the ToL planning task after SSRI treatment, we may thus more comprehensively understand the effect of SSRIs on neural networks during goal-directed planning and identify an early biomarker to potentially improve planning abilities.

Here, we performed a longitudinal fMRI study to investigate the large-scale neural effects of 16 weeks of SSRI treatment on brain network activity during a ToL planning task in medication-free OCD patients. First, we hypothesized that OCD patients, compared to healthy controls (HCs), would show disrupted functional interactions among the DMN, the task-negative network, and the attention or executive control networks (i.e., the dorsal attention network [DAN] and the FPN) in association with planning. Second, we expected that the impaired balance between brain networks would be restored by 16 weeks of SSRI treatment in patients with OCD.

## Results

### Treatment responses and ToL task performance

**Figure 1:**
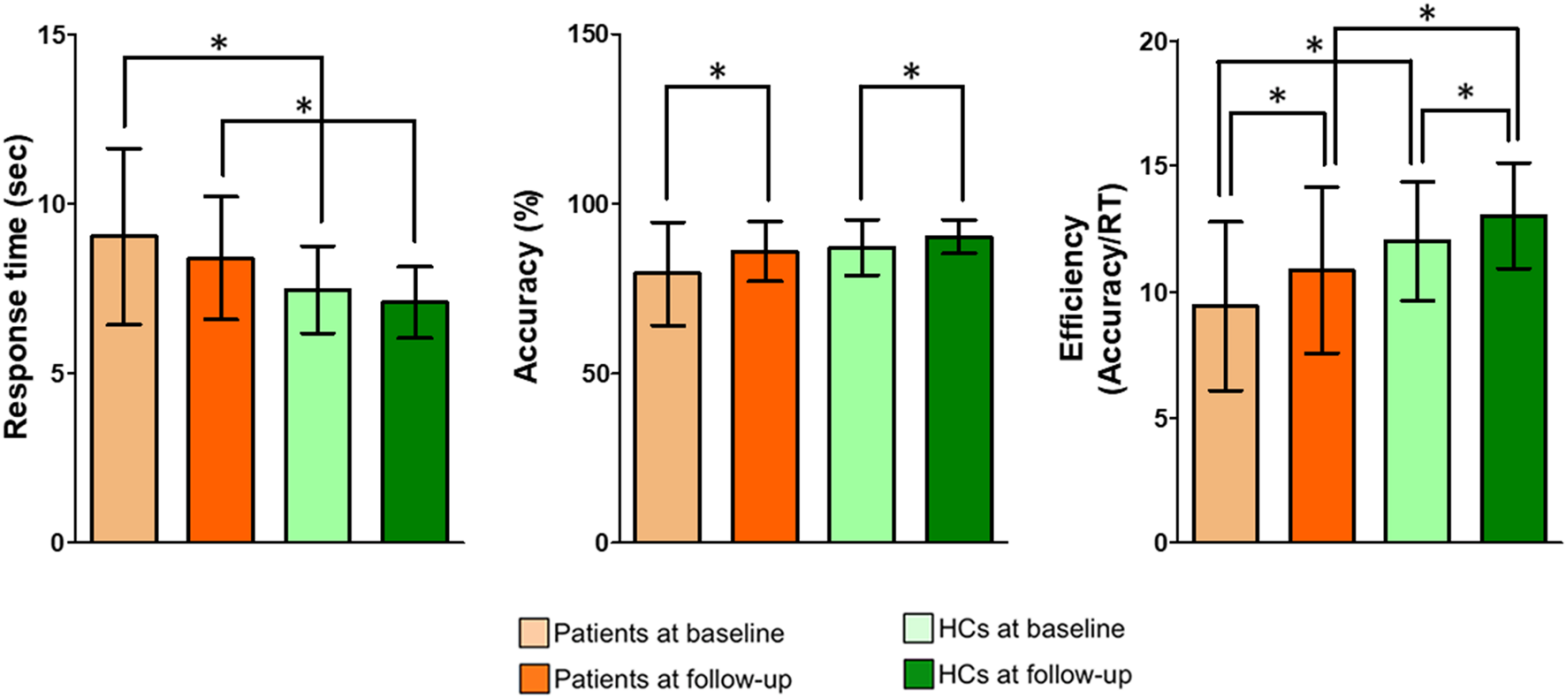
Group comparison of ToL planning task performance. The horizontal lines for each group indicate the means, and the vertical lines for each group indicate the standard deviations. *Indicates that the mean difference was significant at the 0.05 level.

The improvement in clinical symptoms during 16 weeks of treatment in patients with OCD is summarized in Table 1. Comparisons of ToL task performance across the groups and time points are presented in Table 2 and Fig. 1 Additional information is provided in the Supplementary material. Patients with OCD showed a significant improvement in their clinical symptoms after 16 weeks compared to their baseline (all ps < 0.001), showing a 30.4% ± 23.2% reduction in the Yale-Brown Obsessive–Compulsive Scale (Y-BOCS) total score. Regarding the task performance in the ToL condition, both groups showed increased accuracy and enhanced efficiency (defined as the accuracy divided by the response time) at follow-up compared to baseline. However, patients with OCD showed longer response times (RTs) and lower efficiency than HCs at both baseline and follow-up. Detailed analysis results across the task loads are presented in Supplementary Table S2.

**Table 1:** Demographic and clinical characteristics of patients with obsessive–compulsive disorder (OCD) and healthy controls (HCs) at baseline and follow-up.

	OCD (N = 17)	HC (N = 21)	Statistical analysis	*p*		
			χ^2^ or T^a^			
Sex (male/female)	12/5	11/10	1.304	0.254		
Handedness (left/right)	1/16	3/18	0.704	0.401		
Age (years)	26.4 ± 6.0	26.0 ± 5.3	0.225	0.823		
Education years (years)	14.3 ± 2.2	15.4 ± 1.7	− 1.772	0.085		
IQ	112.4 ± 9.6	113.5 ± 10.8	− 0.331	0.742		
Age of onset (years)	16.6 ± 6.03	–	–	–		
Duration of illness (years)	9.9 ± 6.9	–	–	–		
**Comorbidity**^**b**^		–	–	–		
None	8 (47.1)	–	–	–		
Depressive disorder	9 (52.9)	–	–	–		
Bipolar disorder	–	–	–	–		
Personality disorder	–	–	–	–		

	OCD Baseline	OCD Follow-up	Statistical analysis		T^c^	*p*
			χ^2^ or T^a^	*p*		
**Y-BOCS scores**						
Total	30.4 ± 4.1	21.2 ± 7.6	–	–	5.607	< 0.001**
Obsession	16.2 ± 2.1	11.3 ± 4.3	–	–	5.075	< 0.001**
Compulsion	14.2 ± 2.4	10.0 ± 3.6	–	–	5.716	< 0.001**
HAM-D score	11.5 ± 6.2	6.8 ± 5.6	–	–	5.015	< 0.001**
HAM-A score	12.4 ± 8.1	7.2 ± 6.2	–	–	4.703	< 0.001**

Data are shown as the mean ± standard deviation.

*SD* standard deviation, *IQ* intelligence quotient, *Y-BOCS* Yale-Brown Obsessive–Compulsive Scale, *HAM-D* Hamilton Rating Scale for Depression, *HAM-A* Hamilton Rating Scale for Anxiety.

**The mean difference is significant at the 0.005 level.

^a^Independent t-test or Welch's t-test if the variances were not equal; χ^2^ analysis or Fisher's exact test for categorical data.

^b^Number (percentage) of patients who were diagnosed with each comorbid psychiatric disorder.

^c^Paired-samples t-test.

**Table 2:** Behavioral performance results in the Tower of London task in patients with obsessive–compulsive disorder (OCD) and healthy controls (HCs) at baseline and follow-up.

Time point	OCD (N = 17)	HC (N = 21)	Statistical analysis^a^			
			Difference		Z	*p*
**Response time**^**b**^						
Baseline	9.0 ± 2.6	7.5 ± 1.3	Group	Baseline	− 1.982	0.048*
				Follow-up	− 2.246	0.025*
Follow-up	8.4 ± 1.8	7.8 ± 1.1	Time	OCD	− 1.633	0.102
				HC	− 1.512	0.131
**Accuracy**^**c**^						
Baseline	79.3 ± 15.3	87.1 ± 8.2	Group	Baseline	− 1.791	0.073
				Follow-up	− 1.794	0.073
Follow-up	85.8 ± 8.9	90.2 ± 5.0	Time	OCD	− 2.059	0.039*
				HC	− 2.068	0.039*
**Efficiency (Accuracy/RT)**						
Baseline	9.4 ± 3.3	12 ± 2.4	Group	Baseline	− 2.950	0.003**
				Follow-up	− 2.716	0.007*
Follow-up	10.9 ± 3.3	13 ± 2.1	Time	OCD	− 2.911	0.004**
				HC	− 1.999	0.046*

Data are shown as the mean ± standard deviation.

*SD* standard deviation, *RT* response time.

*The mean difference is significant at the 0.05 level.

**The mean difference is significant at the 0.005 level.

^a^Mann–Whitney U test for testing the group difference; Wilcoxon signed-rank test for testing the time difference.

^b^Average RT of correct responses, in second.

^c^Percentage (%) of correct responses.

### Network results

**Figure 2:**
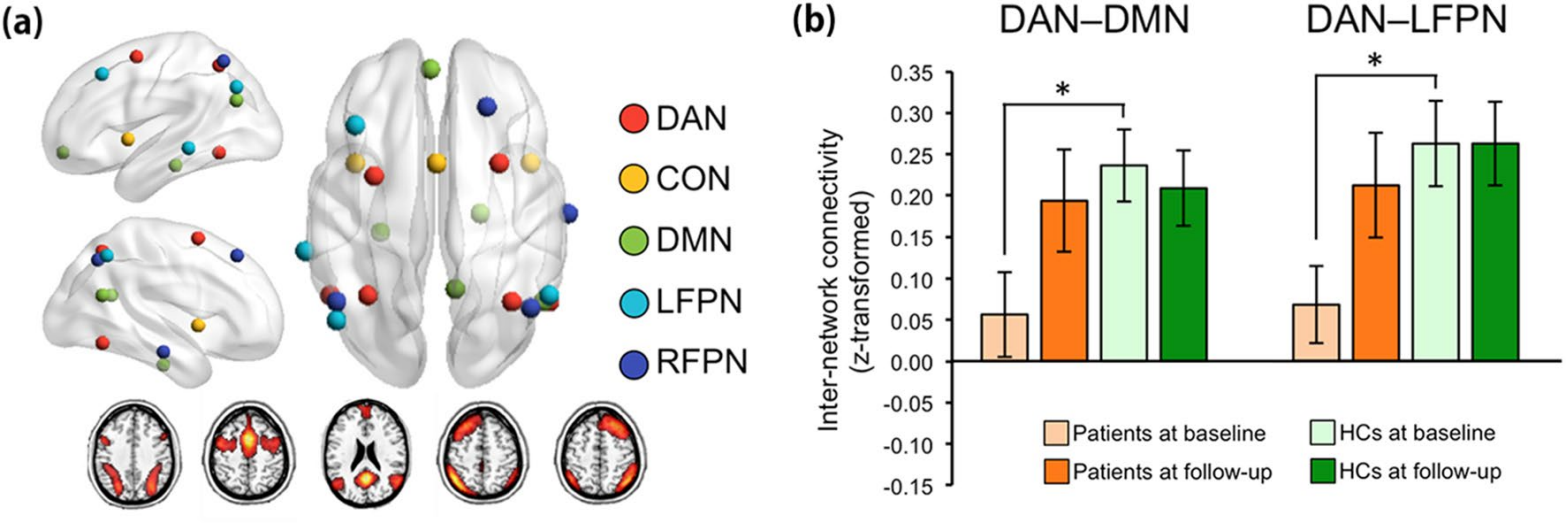
Results from the network analysis using statistical parametric mapping (SPM) 12 software. (**a**) Figures illustrating the location of nodes within each of the functional brain networks, including the dorsal attention network (DAN; red), cingulo-opercular network (CON; yellow), default-mode network (DMN; green), left frontoparietal network (LFPN; sky), and right frontoparietal network (RFPN, blue). The bottom shows activation maps of each component (i.e., functional brain networks) extracted by independent component analysis. (**b**) DAN–DMN and DAN–LFPC internetwork FC during performance of the ToL task. The horizontal lines for each group indicate the means, and the vertical lines for each group indicate the standard deviations. *Indicates that the mean difference is significant at the 0.05 level.

Regarding the fMRI data gathered during the ToL task, the patients exhibited significantly lower baseline internetwork FC than the HCs between the DAN and DMN (t-/q-values = − 2.82/0.04) and between the DAN and LFPN (t-/q-values = − 2.93/0.04) for the contrast of the planning load versus the CC2 load (Fig. 2b). After treatment, these two internetwork FCs increased in patients and showed no significant differences with those of HCs at follow-up (t-/q-values = − 0.21/0.94 and − 0.67/0.85 for DAN–DMN and DAN–LFPN internetwork FCs, respectively). There were no other significant group differences and no significant group by time interaction effects for all other contrasts at baseline and follow-up (all qs > 0.05).

In the exploratory regression analysis, there were no significant correlations between the changes in brain variables showing significant group differences (mentioned above) and the changes in Y-BOCS total score.

## Discussion

This is the first longitudinal study to examine whether internetwork connectivity during the ToL planning task in medication-free patients with OCD could be restored after 16 weeks of SSRI treatment. At baseline, the patients with OCD showed poorer behavioral performance in the ToL task than the HCs. In addition, there were lower levels of DAN–DMN and DAN–LFPN internetwork connectivity during the ToL planning task in the OCD patients than in the HCs. All of the observed dysconnectivity was increased to the corresponding level of the HCs after 16 weeks of SSRI treatment; however, the patients’ behavioral performance in the ToL planning task was not normalized. These findings not only highlight that imbalanced internetwork connectivity underlies the impaired planning capacity of patients with OCD but also suggest that the restoration of altered internetwork connectivity may be an early marker of the SSRI effect on goal-directed planning.

Accumulating evidence has suggested an important role of multiple interactions among different brain networks during executive control processes^25,26^. In particular, it has been suggested that goal-directed planning engages the DAN, FPN, and DMN, and these three networks flexibly interact with one another according to task demands^25,26^. That is, while the DAN is predominantly engaged in visuospatial planning, e.g., while performing the ToL task, the DMN is primarily associated with internally focused planning, such as autobiographical planning. The FPN is activated during both visuospatial and autobiographical planning, suggesting a mediating role between the DAN and DMN^26^. In line with those previous reports, we observed DAN–DMN and DAN–LFPN interactions during the ToL planning task; these interactions were impaired at baseline in patients with OCD compared to HCs. The network dysconnectivity observed in OCD patients may reflect deficient engagement of networks associated with goal-directed planning (i.e., imbalanced connectivity between networks involved in planning). Impaired goal-directed planning in OCD may be due to the use of inappropriate cognitive strategies in solving the ToL problem rather than the impairments in discrete cognitive subcomponents (e.g., attention or memory). Previous studies that suggested inefficient cognitive strategies, such as organization skill, as a main cause of impaired visuospatial memory in OCD further support our interpretation^5,27^. Therefore, the impaired planning capacity found in OCD patients may result from the imbalance between networks associated with planning (i.e., DAN, FPN, and DMN) and related inefficient cognitive strategies.

With regard to impaired planning performance, studies have reported inconsistent results. Several studies suggested impaired planning performance as a trait marker by showing deficient ToL performance in patients’ unaffected first-degree relatives^8,9^ or by showing persistence of impairments after SSRI treatment^15^. In contrast, negative findings from unaffected first-degree relatives of OCD patients^10,11^ and improved planning performance in pediatric OCD patients after CBT^12–14^ suggested that planning capacity is a state marker modifiable by effective treatment. Because the behavioral phenotype is too general to reflect subtle improvements accompanied by complex neural changes during a limited observational period, it has been recommended that neuroimaging biomarkers should be investigated to sensitively detect early potential changes^16,17^. Supporting that recommendation, we found that impaired internetwork connectivity in the OCD patients during the ToL task was restored to the level of HCs after 16 weeks of SSRI treatment, despite the persistent impairment in behavioral performance during the ToL planning task. In addition, although we previously reported that the restoration of the resting-state brain connectome by SSRI treatment was significantly correlated with improvements in OC symptom severity^18^, the same sample in the current study yielded no significant relationship between increased internetwork connectivity during the ToL planning task and symptomatic improvement after SSRI treatment. Thus, the restoration of neural networks during the ToL planning task may not be related to symptomatic improvement; rather, it may be an early marker of the effect of SSRIs specifically on goal-directed planning in patients with OCD.

There are several limitations of this study. First, the sample size used for the final analysis was relatively small due to dropouts at the follow-up scan, which may limit the reproducibility of the results. Second, the improvements in clinical symptoms by SSRI treatment, including OC, depressive, or anxious symptoms, could have been reflected in neural changes detected after SSRI treatment. However, we examined the extent to which these symptomatic improvements were correlated with neural changes and found no significant correlations. Finally, despite the changes in internetwork connectivity after SSRI treatment being assumed to be an early marker of the improvement of planning capacity, behavioral performance during the ToL planning task was not normalized within the study period. This may be the result of an insufficient observational period in this study to result in a behavioral level enhancement of planning performance. Therefore, future studies with larger sample sizes and longer observational periods to reveal improvements in planning performance are needed to confirm the findings of the current study.

In this longitudinal study, we revealed that the reduced internetwork connectivity during the ToL planning task in medication-free OCD patients compared to HCs was restored after 16 weeks of SSRI treatment. Executive functioning such as goal-directed planning is important for a better quality of life as well as daily functioning in patients, and it often takes a considerable amount of time to observe the improvement in cognitive functioning in many psychiatric and neurological disorders. Therefore, the current study results provide valuable guidance for using SSRIs not only for the treatment of OC symptoms but also for the improvement in planning capacity. Furthermore, our findings offer new insight into the altered internetwork connectivity in OCD underlying impaired goal-directed planning as an important pathophysiological mechanism.

## Methods

### Participants

Twenty-six medication-free patients with OCD (9 drug-naïve and 17 unmedicated for more than 1 month) and 26 HCs matched for age, sex, handedness, and intelligence quotient (IQ) participated in this study. At the time of enrollment, fMRI scans during the ToL task were performed, and clinical symptoms were assessed with instruments including the Y-BOCS^28^. Then, patients were provided usual treatment for OCD using an SSRI (i.e., escitalopram with a range of 10–40 mg/day) for 16 weeks in the OCD clinic at Seoul National University Hospital (SNUH). After 16 weeks, 20 OCD patients and 22 HCs completed follow-up clinical assessment and fMRI scans. A total of 10 patients dropped out before the retesting stage for the following reasons: withdrawal of consent in 2 OCD patients, adverse effects in 2 OCD patients, and loss of contact in 2 OCD patients and 4 HCs.

After the fMRI data were preprocessed, 3 patients and 1 HC were excluded due to excessive head motion (> 2.5 mm of translation or 2.5° of rotation and > 0.30 mm for mean framewise displacement [FD])^29^. Therefore, the final sample of participants with both pre- and posttreatment scans consisted of 17 OCD patients (7 drug-naïve and 10 medication-free for more than 1 month at baseline) and 21 matched HCs. The demographic and clinical information of both groups is summarized in Table 1. Additional information about our participants is available in the Supplementary material.

This study was conducted in accordance with the Declaration of Helsinki and was approved by the Institutional Review Board of SNUH (IRB no. H-1002-035-309). Written informed consent was obtained from all of the participants after a full explanation of the study procedure was provided.

### Tower of london task

**Figure 3:**
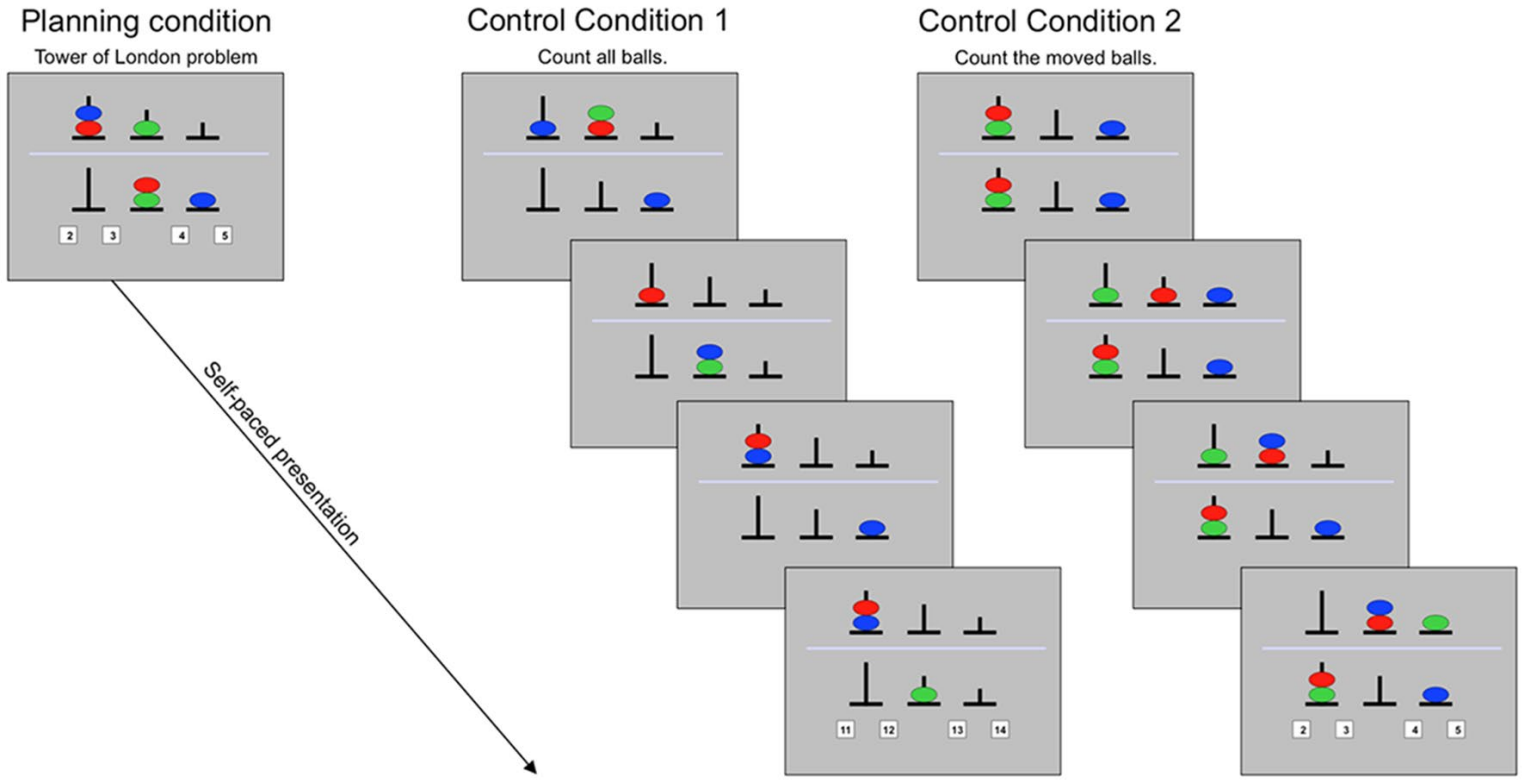
Depiction of the task paradigm. In the planning (Tower of London) condition, participants were asked to calculate the minimum number of moves necessary to achieve the goal configuration (in the lower half of the display) by moving balls, one at a time, from the starting configuration (in the upper half). In control condition 1 (CC1), participants were asked to count all balls presented across all pictures. In control condition 2 (CC2), participants were asked to count the moved balls across all pictures. For all task conditions, participants had to press the button corresponding to the number of correct answers for each trial.

We used a modified version of the ToL task adapted from^30^, and details regarding the task procedures are provided in the Supplementary material. The task consisted of 3 conditions, including a planning condition with the ToL problem and two control conditions (Fig. 3). In the planning condition, the participants were asked to mentally calculate the minimum number of moves necessary to reach the goal configuration (located in the lower half of the display) by moving one ball at a time from the starting configuration (in the upper half). In the first control condition (CC1), the participants were asked to count the number of balls presented in a series of pictures. CC1 was designed to control for visuospatial information processing and working memory demands. In the second control condition (CC2), participants saw a sequence of moving balls that was the backward sequence of the solution to the ToL problem, essentially showing the participants how to solve the ToL problem in their minds using the method of “backward search”^31^. CC2 was designed to control for visuospatial processing and potential anticipatory processes but was not related to planning per se. At the end of all 3 conditions, the participants had to press a button corresponding to four possible answers.

The task consisted of 3 runs with 105 trials (35 trials [15 planning, 9 CC1, and 11 CC2] per run) and was presented in a pseudorandomized self-paced design. All 3 task conditions were presented as separate blocks in each run to control for task switching effects. Each task condition included 4 difficulty levels resulting in different levels of cognitive loading (i.e., load levels). All participants successfully executed practice trials before participating in the fMRI scan. Behavioral performance during the ToL task was measured by response time (RT; i.e., the average RT of correct responses in sec) and accuracy (i.e., percentage of correct responses) for each task condition and load level. These two variables were computed by collapsing and averaging across all four load levels of each task condition to simplify the data. In addition, performance efficiency was defined as the accuracy divided by the RT in order to correct the confounding effect of response time on accuracy.

### Image acquisition and preprocessing

Image data were collected on a 3T scanner (Siemens Magnetom Trio, Erlangen, Germany) using a T2*-weighted gradient echo-planar imaging (EPI) sequence during the ToL task. For image preprocessing, statistical analyses, and creation of Fig. 2a and supplementary Figs. S1–S3, we used statistical parametric mapping (SPM) 12 software (http://www.fil.ion.ucl.ac.uk/spm). Details regarding image acquisition and preprocessing are provided in the Supplementary material.

### Construction of functional brain networks by independent component analysis

Independent component analysis (ICA) is a data-driven method that employs no a priori assumptions about the nature of task-evoked blood oxygenation level-dependent (BOLD) responses and ensures that the network definitions by ICA are not biased by a priori selection of specific seed regions (nodes) or networks. We performed spatial ICA across all preprocessed data obtained during the ToL task (pooled across groups and time points) to identify spatially independent, temporally coherent functional networks using the Group ICA for fMRI Toolbox (GIFT; http://mialab.mrn.org/software/gift/). Briefly, the number of estimated independent components (IC) was based on the minimum description length (MDL) criteria^32^, which resulted in 45 ICs. We used the Infomax algorithm with 10 repetitions of ICASSO to ensure stability of the decomposed components^33^. Subject-specific ICA spatial maps and corresponding time courses were computed by back-reconstruction using spatiotemporal (dual) regression^34^. More detailed descriptions for ICA can be found elsewhere^35^. The spatial anatomy of each component was identified using one-sample t-tests (Fig. 2a). These spatial maps were compared with known atlases and prior literature^26,36,37^ to identify the five main networks associated with external attention and cognitive control^26,38^, including the DAN, cingulo-opercular network (CON), left FPN (LFPN), right FPN (RFPN), and DMN. We confirmed that these networks were more related to the ToL task events than other ICs using temporal sorting with a general linear model (GLM) design matrix (with regressors for the task events, error trials, and motion parameters; see Standard univariate fMRI analysis in the Supplementary material for details) as implemented in GIFT. When the resulting ICs were sorted according to their R-squared statistic, the DAN showed the highest R-squared value for the ToL condition.

### Network analysis

To estimate the strength of FC between functional brain networks according to the task condition, we performed a network analysis. The nodes for each of the five networks consisted of 5-mm radius spheres centered on the MNI coordinates of all significant clusters in the statistically thresholded component spatial maps (Supplementary Table S1). The strengths of FC between nodes were estimated using a correlational psychophysiological interaction (cPPI; https://www.nitrc.org/projects/cppi_toolbox/). The cPPI measures are context-dependent FC measures based on partial associations to isolate covariations in the task-related modulations of neural activity as distinct from the task-unrelated responses, noise, and coactivation effects^38^. For the cPPI analysis, we designed a new GLM matrix [with regressors including task events, error trials, motion parameters, the mean white matter (WM) signal, and the mean cerebrospinal fluid (CSF) signal] for each subject. To normalize the variance in the correlation values, all values in the subject-specific FC matrices were converted to z-values using Fisher's r-to-z transformation. We then computed the average connectivity (mean Fisher z-value) across all node-to-node connections between two networks as internetwork connectivity. For exploratory purposes, we also computed the average connectivity across node pairs within the same network as intranetwork connectivity.

### Standard univariate fMRI analysis

Though the aim of this study was to test network connectivity, we also performed an exploratory, univariate fMRI analysis due to its value as the gold standard for task-fMRI data and to test the association between the magnitude of task-induced activation and SSRI administration. Details regarding the standard univariate fMRI analysis methods, results, and discussion are provided in the Supplementary material (Supplementary material and Methods; Supplementary material; Supplementary Discussion; Supplementary Figs. S1–S3).

### Statistical analysis

SPSS software ver. 23.0 (IBM Corp., Armonk, NY, USA) was used for the statistical analyses. Significance levels were set at *p* < 0.05. Because the behavioral data from the ToL task performance were not normally distributed, Mann–Whitney U tests and Wilcoxon signed-rank tests were used to examine differences between the two groups and between the two scan times. For other variables, two-sample t-tests and paired t-tests were used to examine differences between the two groups at each scan time and differences between the two scan times in each group. To test a group by time interaction effect, we performed 2 (group) by 2 (time) repeated measures analysis of variance. We also computed the values of changes (subtracting baseline from follow-up) for all values from the network analysis. The aforementioned statistical analyses were then performed with all these estimated changes and differences. Significance levels for the network analysis were set at q < 0.05 (i.e., false discovery rate [FDR]-adjusted *p* < 0.05). Exploratory regression analyses were performed to test associations between changes in brain responses and the Y-BOCS total score over 16 weeks.

## Supplementary information

Supplementary Information.

## Data Availability

The data that support the findings of this study are available from the corresponding author upon reasonable request.
