# Supplementary material for: The effects of selective serotonin reuptake inhibitors on brain functional networks during goal-directed planning in obsessive–compulsive disorder
Source: Sci Rep. 2020 Nov 26;10:20619. doi: 10.1038/s41598-020-77814-4 (PMC7691328; doi:10.1038/s41598-020-77814-4)
Supplement: Supplementary file 1 — Supplementary Information. [file 41598_2020_77814_MOESM1_ESM.docx]

**The effects of selective serotonin reuptake inhibitors on brain functional networks during goal-directed planning in obsessive-compulsive disorder**

**Supplementary Materials**

Minah Kim^1,2,#^, Wi Hoon Jung^3,#^, Geumsook Shim^4^, Jun Soo Kwon^1,2,5,*^

^1^Department of Neuropsychiatry, Seoul National University Hospital, Seoul, Republic of Korea

^2^Department of Psychiatry, Seoul National University College of Medicine, Seoul, Republic of Korea

^3^Department of Psychology, Daegu University, Gyeongsan, Republic of Korea

^4^KAIST Clinic Pappalardo Center, KAIST, Daejeon, Republic of Korea

^5^Institute of Human Behavioral Medicine, SNU-MRC, Seoul, Republic of Korea

^#^These authors contributed equally to this work as joint first authors.

**^*^Corresponding Author**

Jun Soo Kwon, MD, PhD

Department of Psychiatry, Seoul National University College of Medicine, 101 Daehak-no, Chongno-gu, Seoul, Republic of Korea 03080

Tel.: +82 2 2072 2972

Fax: +82 2 747 9063

E-mail: kwonjs@snu.ac.kr

**1. Supplementary Methods**

***1.1. Participants***

Twenty-six medication-free patients with obsessive-compulsive disorder (OCD; 9 drug naïve and 17 unmedicated for more than 1 month) were recruited from the OCD clinic at Seoul National University Hospital (SNUH). All patients were evaluated by certified psychiatrists using the Structured Clinical Interview for DSM-IV Axis I Disorders (SCID-1) and fulfilled the criteria for OCD. We also used internet advertisements to recruit 26 healthy controls (HCs) who were matched to the patients by age, sex, handedness, and intelligence quotient (IQ). The absence of axis I psychiatric disorders in HCs was confirmed using the Structured Clinical Interview for the DSM-Non-Patient edition (SCID-NP). For all participants, the exclusion criteria were a lifetime history of psychotic disorder, bipolar disorder, substance abuse/dependence (except nicotine), Tourette’s disorder, or other tic-related conditions as well as a history of seizure, head injury, or mental retardation (IQ < 70).

At the time of enrollment, functional magnetic resonance imaging (fMRI) scans were taken during performance of the Tower of London (ToL) task. Participants were assessed for their severity of obsessive-compulsive, depressive, and anxious symptoms using the Yale-Brown Obsessive Compulsive Scale (Y-BOCS)^1^, the Hamilton Rating Scale for Depression (HAM-D)^2^, and the Hamilton Rating Scale for Anxiety (HAM-A)^3^, respectively. Then, the patients were provided usual treatment for OCD with a selective serotonin reuptake inhibitor (SSRI; i.e., escitalopram, which ranged from 10-40 mg/day) for 16 weeks in the OCD clinic at SNUH. Two patients were concurrently prescribed clonazepam, either 0.5 or 0.75 mg/day, and the remaining patients used only escitalopram. None of the patients were taking antipsychotics or mood stabilizers or receiving cognitive-behavioral therapy (CBT) or psychoanalytic psychotherapy during the study period. After 16 weeks, 20 patients with OCD and 22 HCs completed the follow-up clinical assessment and fMRI scans. The mean interval (standard deviation) between the baseline and follow-up assessments was 120.9 (16.1) days for patients and 122.1 (23.2) days for controls (controls versus patients: t = 0.18; p = 0.86). A total of 10 patients dropped out before the retesting stage for the following reasons: withdrawal of consent in 2 OCD patients, adverse effects in 2 OCD patients, and loss of contact in 2 OCD patients and 4 HCs.

After the fMRI data were preprocessed, 3 patients and 1 HC were excluded due to excessive head motion (> 2.5 mm of translation or 2.5° of rotation and > 0.30 mm for mean framewise displacement [FD])^4^. Therefore, the final sample of participants with both pre- and posttreatment scans consisted of 17 OCD patients (7 drug naïve and 10 medication-free for more than 1 month at the baseline) and 21 matched HCs. The demographic and clinical information of each group is summarized in Supplementary Table S1. Eight patients were free of comorbid axis I psychiatric disorders, and 9 had the following axis I psychiatric comorbidities: depressive disorder not otherwise specified (n = 6) and dysthymic disorder (n = 3). None of the patients had comorbid axis II psychiatric disorders.

***1.2. Tower of London Task***

We used a modified version of the ToL task adapted from Wagner et al.^4^. The task consisted of 3 conditions, including a planning condition with the ToL problem and two control conditions. In the planning condition, participants were shown a picture with two configurations of 3 colored balls (red, green, and blue) on 3 vertical rods of different heights, which could accommodate 1, 2, or 3 balls each. Participants were asked to mentally calculate the minimum number of moves necessary to reach the goal configuration (located in the lower half of the display) by moving one ball at a time from the starting configuration (in the upper half). They were required to press the button corresponding to the number of moves needed to solve the problem from among the four possible answers (2, 3, 4, and 5) presented beneath the goal configuration. In the first control condition (CC1), participants were shown a series of 2 to 5 pictures and were asked to count the number of balls (ranging from 11 to 14) presented across all pictures. CC1 was designed to control for visuospatial information processing and working memory demands. In the second control condition (CC2), participants were shown 2 to 5 balls moving in the reverse sequence of the solution of the ToL problem, essentially showing the participants how to solve the ToL problem in their minds using the method of “backward search”^5^. In contrast to the planning condition, the goal configuration in the first picture for each CC2 trial was the same as the starting configuration. Participants were asked to count the number of moving balls. CC2 was designed to control for visuospatial processing and potential anticipatory processes (i.e., participants can anticipate the rod that the next ball will move to) that were not related to planning per se. The last pictures for CC1 and CC2 contained four possible answers. Participants were required to press the button corresponding to the correct answer for each trial.

The task consisted of 3 runs with a total of 105 trials (35 trials [15 planning, 9 CC1, and 11 CC2] per run) and used a pseudorandomized self-paced design. The 3 task conditions were presented as separate blocks in each run to control for task-switching effects. For example, after all trials for the planning condition had been presented, a trial for the CC1 was presented. Each task condition included 4 difficulty levels resulting in different levels of cognitive loading (i.e., load levels). For the planning condition, each trial consisted of a ToL problem (a maximum time of 15 sec for easy problems with 2 and 3 moves and of 30 sec for difficult problems with 4 and 5 moves) and a fixation baseline (at least 6 sec). After participants pressed one of the 4 buttons, the picture with the ToL problem disappeared, and a fixation cross was presented for the remaining length of the trial. A run contained 5 ToL problems with 2 moves, 4 with 3 moves, 3 with 4 moves, and 3 with 5 moves. In the CC1 condition, each picture was presented for 2000 msec with an interstimulus interval (ISI) of 200 msec. The last picture with 4 possible answers was presented for 5 sec. In the CC2 condition, each picture was presented for 1000 msec (for pictures in which 2 or 3 balls moved) or 1300 msec (for pictures in which 4 or 5 balls moved) with an ISI of 0 msec to obtain the effect of the ball moving. The last picture with 4 possible answers was presented for 3 sec. For both the CC1 and CC2 conditions, the fixation baseline was presented for at least 6 sec. The numbers of trials at the 4 load levels were as follows: 3 at level 1, 2 at level 2, 2 at level 3, and 2 at level 4 for the CC1 condition; 4 at level 1, 3 at level 2, 2 at level 3, and 2 at level 4 for the CC2 condition. The different numbers of trials at different load levels were used to achieve a comparable number of acquired scans and comparable statistical power across the categories and the conditions^6^. All participants successfully executed practice trials before participating in the fMRI scan.

Behavioral performance during the ToL task was measured by reaction time (RT, specifically, the average RT in seconds for correct responses) and accuracy (i.e., the percentage of correct responses) for each task condition and load level. These two variables were computed by collapsing and averaging across all four load levels of each task condition to simplify the data. In addition, performance efficiency was calculated as accuracy divided by RT.

***1.3. Image Acquisition and Preprocessing***

Image data were collected on a 3 T scanner (Siemens Magnetom Trio, Erlangen, Germany) using a T2*-weighted gradient echo-planer imaging (EPI) sequence during the ToL task. The parameters for the task fMRI data were echo time (TE) = 30 msec, repetition time (TR) = 2 sec, flip angle (FA) = 90°, voxel size = 3.4 × 3.4 × 4.0 mm^3^, 27 interleaved axial slices, and 360 volumes per run. High-resolution T1-weighted magnetization-prepared rapid-gradient echo anatomical images were also obtained (TE = 1.89 msec, TR = 1.670 msec, FA = 9°, voxel size = 1.0 × 0.98 × 0.98 mm^3^, and 208 sagittal slices).

For the image preprocessing, statistical analyses, and creation of figures, we used SPM12 software (http://www.fil.ion.ucl.ac.uk/spm). After the first 4 volumes were discarded, data were corrected for both slice timing acquisition and head motion and spatially normalized to the MNI reference brain. Then, the data were smoothed with a 6-mm full width at half-maximum (FWHM) Gaussian kernel.

***1.4. Standard Univariate fMRI Analysis***

At the first level, preprocessed data were analyzed in the context of the GLM consisting of separate regressors for trials of each task condition (ToL, CC1, and CC2) and 4 load levels to compute the overall mean brain response during task performance and the brain response according to a linearly increasing task load for each task condition^7^. All task trials with error trials and motion parameters (as regressors of no interest) were convolved with the canonical hemodynamic response function as implemented in SPM. Weighted contrasts were estimated for the main effects of task condition (ToL condition versus one of the control conditions) and task load (ToL level versus the load of one of the control conditions). These contrast images were used for second-level (random-effects) analyses.

**2. Supplementary Results**

***2.1. Treatment Response and ToL Task Performance***

The patients with OCD showed a significant improvement in their clinical symptoms after 16 weeks compared to their baseline (all ps < 0.001), showing a 30.4% ± 23.2% reduction in Y-BOCS total scores. The patients also showed significant reductions in HAM-D (51.5% ± 35.0%) and HAM-A scores (52.0% ± 33.6%) at follow-up (p < 0.001).

The task performance results for each group are presented in Supplementary Table S2. Significant time and group differences were mostly found in the ToL and CC2 conditions, although some differences were observed in the CC1 condition. For example, both groups showed decreased RT and increased accuracy in the ToL condition at follow-up compared to baseline (p-value = 0.02 and 0.02 for the RT of level 1 in the patients and in HCs, respectively; p-value = 0.04 and 0.04 for the overall accuracy level in the patients and in HCs, respectively). However, the patients showed longer RTs than the HCs for different ToL load levels at both baseline and follow-up (p-value = 0.03, 0.02, 0.03, and 0.05 for level 1, level 2, level 3, and overall level at baseline, respectively; p-value = 0.03, 0.02, 0.03, and 0.03 for level 1, level 3, level 4, and overall level at follow-up, respectively). The patients also had less accuracy than the HCs in the ToL condition (p-value = 0.01 for level 4 at baseline). In the CC2 condition, the patients also showed longer RTs and lower accuracy than the HCs at both baseline and follow-up (p-values = 0.03, 0.01, 0.01, 0.01, and 0.01 for the RTs for level 1, level 2, level 3, level 4, and overall level, respectively, at baseline; p-values = 0.04 and 0.04 for the RT of level 3 and overall level, respectively, at follow-up; p-values = 0.02 and 0.05 for the accuracy of levels 1 and 2, respectively, at baseline; p-value = 0.05 for the accuracy of level 4 at follow-up). Group differences in changes (follow-up minus baseline) were observed for the RTs in CC1 level 2 and the accuracy in ToL level 4 and CC2 level 4.

Significant group differences in the efficiency score (accuracy/RT) were observed primarily in the ToL and CC2 conditions, although a group difference was also observed in CC1 level 4. Both groups showed significantly increased efficiency scores in the ToL condition at follow-up compared to baseline (p-values = 0.01, 0.02, and 0.01 for level 1, level 2, and overall level, respectively, in patients; p-values = 0.01 and 0.05 for level 1 and overall level, respectively, in HCs). At both baseline and follow-up, the patients had lower efficiency scores than the HCs in the ToL condition (p-values = 0.01, 0.01, 0.01, 0.02, and 0.01 for level 1, level 2, level 3, level 4, and overall level, respectively, at baseline; p-values = 0.02, 0.01, and 0.01 for level 1, level 3, and overall level, respectively, at follow-up) and in the CC2 condition (p-values = 0.02, 0.01, 0.01, and 0.01 for level 1, level 2, level 3, and overall level, respectively, at baseline; p-values = 0.03 and 0.05 for level 2 and overall level, respectively, at follow-up). Changes (follow-up minus baseline) were not significantly different between the patients and HCs.

***2.2. Standard Univariate GLM Results***

The main effects of task and load are presented in Supplementary Figure S1. Consistent with previous findings^8, 9^, planning (compared with control tasks) was related to increased activation in the DLPFC, premotor cortex, and visuospatial areas (precuneus, superior/inferior parietal and superior occipital cortices) across groups. Increasing task load during the planning task was correlated with increased activation in the DLPFC, premotor, precuneus, striatum, superior/inferior parietal cortex, anterior insula and cingulate cortices, and visual areas. When contrast images at follow-up were compared with those at baseline for each group, one significant cluster was found in HCs: HCs showed greater activation in the right supramarginal cortex (x, y, z = 60, -27, 39; t-/z-value = 4.70/4.12) at follow-up than at baseline for the contrast of the planning task versus the CC1 task (Supplementary Figure S2A). However, there were no significant group × time interaction effects for any of the contrast images.

Two-sample t-tests revealed that the patients had less activation than the HCs at baseline in the right precuneus/paracentral lobule (x, y, z = 6, -51, 45; t-/z-value = 4.26/3.81) for the contrast of the planning task versus the CC2 task (Supplementary Figure S2B) and greater activation in the right postcentral cortex (x, y, z = 18, -36, 60; t-/z-value = 5.27/4.51), superior parietal cortex (x, y, z = 24, -57, 66; t-/z-value = 4.57/4.03), and middle temporal cortex (x, y, z = 57, -63, 0; t-/z-value = 4.56/4.02) for the contrast of the planning load versus the CC2 load (Supplementary Figure S2C). However, there were no significant group differences at follow-up.

Multiple regression analysis at the region-of-interest (ROI) level for the abovementioned regions did not reveal any significant association with symptom severity. However, exploratory whole-brain voxel-level analysis revealed one significant cluster: decreases in patients’ OC symptom severity (change in Y-BOCS total scores) after 16 weeks were associated with increases in activation in the left calcarine cortex (x, y, z = -18, -72, 15; t-/z-value = 7.88/4.88) for the contrast of the planning task versus the CC1 task (Supplementary Figure S3).

**3. Supplementary Discussion**

In the present study, standard univariate GLM analyses revealed that patients with OCD had less activation than the HCs in the precuneus during planning across difficulty levels and greater activation in the parietal and temporal cortices with increasing task load. A decreased level of recruitment of the precuneus in patients has been repeatedly reported in previous studies^7, 10, 11^. The precuneus has been implicated not only in episodic memory retrieval and self-processing operations but also in visuospatial imagery^12^. The reduced neural responsiveness in the precuneus may reflect a deficient engagement in visuospatial imagery. In contrast, increased recruitment in parietal and temporal areas with increasing task load in patients may reflect increased engagement of cognitive functions associated with these regions. Functional abnormalities in the parietal areas in OCD have been observed during various cognitive tasks, such as biological motion perception^13^ and reversal learning^14^. The superior parietal and posterior middle temporal cortices are involved in the processing of visuospatial information. Considering the parietal activation, it is conceivable that the patients may have used arithmetic processing, nonverbal memory, or sustained attention, which are involved in the ToL task, more than the HCs. Although we used specific control conditions to remove such confounding effects, those effects could not be completely ruled out. Consistent with our findings, previous longitudinal studies in pediatric OCD patients receiving CBT have also found increased activation in the frontal and parietal regions with increasing task load at baseline, and this increased activation was normalized after CBT^15, 16^.

**References**

**1.** Goodman WK, Price LH, Rasmussen SA, Mazure C, Fleischmann RL, Hill CL, Heninger GR, Charney DS. The Yale-Brown obsessive compulsive scale. I. Development, use, and reliability. *Arch Gen Psychiatry* Nov 1989;46(11):1006-1011.

**2.** Hamilton M. A rating scale for depression. *J Neurol Neurosurg Psychiatry* Feb 1960;23:56-62.

**3.** Hamilton M. The assessment of anxiety states by rating. *Br J Med Psychol* 1959;32(1):50-55.

**4.** Power JD, Barnes KA, Snyder AZ, Schlaggar BL, Petersen SE. Spurious but systematic correlations in functional connectivity MRI networks arise from subject motion. *Neuroimage* Feb 1 2012;59(3):2142-2154.

**5.** Mayer RE. *Thinking, problem solving, cognition*. second ed. New York, NY: Freeman; 1992.

**6.** Wagner G, Koch K, Reichenbach JR, Sauer H, Schlosser RG. The special involvement of the rostrolateral prefrontal cortex in planning abilities: an event-related fMRI study with the tower of London paradigm. *Neuropsychologia* 2006;44(12):2337-2347.

**7.** Van den Heuvel OA, Veltman DJ, Groenewegen HJ, Cath DC, Van Balkom AJ, Van Hartskamp J, Barkhof F, Van Dyck R. Frontal-striatal dysfunction during planning in obsessive-compulsive disorder. *Arch Gen Psychiatry* Mar 2005;62(3):301-309.

**8.** Newman SD, Greco JA, Lee D. An fMRI study of the tower of London: a look at problem structure differences. *Brain Res* Aug 25 2009;1286:123-132.

**9.** Van den Heuvel OA, Groenewegen HJ, Barkhof F, Lazeron RH, van Dyck R, Veltman DJ. Frontostriatal system in planning complexity: a parametric functional magnetic resonance version of tower of London task. *Neuroimage* Feb 2003;18(2):367-374.

**10.** Vaghi MM, Vertes PE, Kitzbichler MG, et al. Specific frontostriatal circuits for impaired cognitive flexibility and goal-directed planning in obsessive-compulsive disorder: evidence from resting-state functional connectivity. *Biol Psychiatry* Apr 15 2017;81(8):708-717.

**11.** Van den Heuvel OA, Mataix-Cols D, Zwitser G, Cath DC, Van der Werf YD, Groenewegen HJ, Van Balkom AJ, Veltman DJ. Common limbic and frontal-striatal disturbances in patients with obsessive compulsive disorder, panic disorder and hypochondriasis. *Psychol Med* Nov 2011;41(11):2399-2410.

**12.** Cavanna AE, Trimble MR. The precuneus: a review of its functional anatomy and behavioural correlates. *Brain* 01/01 2006;129(Pt 3):564-583.

**13.** Jung WH, Gu BM, Kang DH, Park JY, Yoo SY, Choi CH, Lee JM, Kwon JS. BOLD response during visual perception of biological motion in obsessive-compulsive disorder: an fMRI study using the dynamic point-light animation paradigm. *Eur Arch Psychiatry Clin Neurosci* Feb 2009;259(1):46-54.

**14.** Chamberlain SR, Menzies L, Hampshire A, et al. Orbitofrontal dysfunction in patients with obsessive-compulsive disorder and their unaffected relatives. *Science* Jul 18 2008;321(5887):421-422.

**15.** Huyser C, Veltman DJ, Wolters LH, De Haan E, Boer F. Functional magnetic resonance imaging during planning before and after cognitive-behavioral therapy in pediatric obsessive-compulsive disorder. *J Am Acad Child Adolesc Psychiatry* Dec 2010;49(12):1238-1248.e1231-1235.

**16.** Van der Straten A, Huyser C, Wolters L, Denys D, Van Wingen G. Long-term effects of cognitive behavioral therapy on planning and prefrontal cortex function in pediatric obsessive-compulsive disorder. *Cognit Neurosci Neuroimaging* Apr 2018;3(4):320-328.

**Supplementary Tables**

**Supplementary Table S1.** Nodes comprising functional brain networks used.

| Region | MNI coordinate (x, y, z) |
| --- | --- |
| Dorsal Attention Network (DAN) | |
| R.IPC | 36, -60, 51 |
| L.IPC | -33, -57, 51 |
| L.DLPFC | -30, 0, 57 |
| R.DLPFC | 30, 6, 60 |
| R.ITC | 54, -60, -12 |
| L.ITC | -51, -57, -9 |
| Cingulo-Opercular Network (CON) | |
| ACC | 0, 6, 63 |
| L.AIS | -39, 6, 0 |
| R.AIS | 45, 6, 0 |
| Left Frontoparietal Network (LFPN) | |
| L.IPC | -48, -69, 36 |
| L.DLPFC | -39, 24, 45 |
| L.MTC | -63, -36, -6 |
| R.IPC | 54, -57, 48 |
| Right Frontoparietal Network (RFPN) | |
| R.IPC | 45, -63, 45 |
| R.DLPFC | 24, 33, 48 |
| R.MTC | 64, -18, -18 |
| L.IPC | -48, -60, 54 |
| Default Mode Network (DMN) | |
| PCC | 9, -54, 21 |
| MPFC | -3, 51, -9 |
| L.IPC | -48, -69, 27 |
| R.IPC | 51, -60, 21 |
| L.Hippocampus | -27, -27, -18 |
| R.Hippocampus | 21, -18, -27 |

Abbreviations: R, right; L, left; IPC, inferior parietal cortex; DLPFC, dorsolateral prefrontal cortex; ITC, inferior temporal cortex; ACC, anterior cingulate cortex; AIS, anterior insula; MTC, middle temporal cortex; PCC, posterior cingulate cortex; MPFC, medial prefrontal cortex.

**Supplementary Table S2.** Behavioral data from the Tower of London (ToL) and two control tasks (CC1 and CC2).

| Task  condition | Difficulty  level | Patients | |  | Healthy controls (HCs) | |
| --- | --- | --- | --- | --- | --- | --- |
|  |  | Baseline | Follow-up |  | Baseline | Follow-up |
|  |  | mean (SD) | mean (SD) |  | mean (SD) | mean (SD) |
| Response time (RT; average RT of correct responses, in sec) | | | | | | |
| ToL | Level 1 | 5.59 (1.47)*^a^ | 4.88 (1.08)*^b,c^ |  | 4.54 (0.95) | 4.24 (0.65)*^d^ |
|  | Level 2 | 6.95 (1.58)*^a^ | 6.23 (1.51)*^c^ |  | 5.79 (0.93) | 5.70 (1.05) |
|  | Level 3 | 10.85 (3.41)*^a^ | 9.92 (2.97)*^b^ |  | 8.60 (1.93) | 7.77 (1.40) |
|  | Level 4 | 12.74 (4.78) | 12.52 (3.11)*^b^ |  | 10.91 (2.74) | 10.62 (2.31) |
|  | Overall level | 9.03 (2.60)*^a^ | 8.39 (1.82)*^b^ |  | 7.46 (1.29) | 7.08 (1.05) |
| CC1 | Level 1 | 4.42 (0.58) | 4.39 (0.43) |  | 4.40 (0.32) | 4.28 (0.38) |
|  | Level 2 | 6.81 (0.65) | 6.71 (0.36) |  | 6.73 (0.39) | 6.94 (0.40)*^d,e^ |
|  | Level 3 | 9.18 (0.85) | 9.11 (0.58) |  | 9.05 (0.49) | 9.04 (0.47) |
|  | Level 4 | 11.41 (0.95) | 11.64 (0.57) |  | 11.51 (0.41) | 11.57 (0.45) |
|  | Overall level | 7.95 (0.71) | 7.96 (0.44) |  | 7.92 (0.30) | 7.96 (0.35) |
| CC2 | Level 1 | 3.48 (0.54)*^a^ | 3.39 (0.45) |  | 3.22 (0.30) | 3.21 (0.28) |
|  | Level 2 | 4.47 (0.52)*^a^ | 4.41 (0.41) |  | 4.25 (0.23) | 4.26 (0.17) |
|  | Level 3 | 6.71 (0.68)*^a^ | 6.71 (0.48)*^b^ |  | 6.43 (0.27) | 6.41 (0.18) |
|  | Level 4 | 7.82 (0.78)*^a^ | 7.78 (0.51) |  | 7.58 (0.30) | 7.61 (0.24) |
|  | Overall level | 5.63 (0.59)*^a^ | 5.57 (0.44)*^b^ |  | 5.37 (0.19) | 5.38 (0.18) |
| Accuracy (% of correct responses) | | | | | | |
| ToL | Level 1 | 87.84 (11.60) | 93.32 (9.14) |  | 91.74 (9.16) | 97.46 (4.46)*^d^ |
|  | Level 2 | 82.84 (15.45) | 87.26 (14.76) |  | 87.70 (10.75) | 88.89 (6.65) |
|  | Level 3 | 72.57 (21.91) | 75.19 (14.44) |  | 78.32 (17.38) | 84.14 (9.66) |
|  | Level 4 | 73.87 (23.55)*^a^ | 87.59 (17.05)*^c^ |  | 90.49 (11.79) | 90.49 (11.79)*^e^ |
|  | Overall level | 79.27 (15.25) | 85.84 (8.85)*^c^ |  | 87.06 (8.21) | 90.24 (5.00)*^d^ |
| CC1 | Level 1 | 98.04 (4.36) | 94.12 (12.48) |  | 94.71 (7.54) | 96.30 (8.83) |
|  | Level 2 | 97.05 (6.56) | 96.08 (9.37) |  | 94.44 (9.63) | 92.85 (13.51) |
|  | Level 3 | 88.22 (17.45) | 92.14 (8.59) |  | 94.44 (9.63) | 91.27 (12.49) |
|  | Level 4 | 85.28 (11.60) | 90.19 (11.87) |  | 85.71 (15.16) | 90.47 (16.31) |
|  | Overall level | 92.16 (5.87) | 93.14 (6.88) |  | 92.33 (5.87) | 92.72 (9.83) |
| CC2 | Level 1 | 96.58 (5.15)*^a^ | 95.59 (12.54) |  | 99.60 (1.81) | 98.41 (4.27) |
|  | Level 2 | 92.82 (9.57)*^a^ | 95.43 (6.86) |  | 97.36 (6.94) | 97.89 (5.68) |
|  | Level 3 | 96.08 (9.37) | 93.14 (16.73) |  | 98.41 (5.02) | 97.61 (5.99) |
|  | Level 4 | 99.02 (4.05) | 97.05 (6.56)*^b^ |  | 96.82 (6.72) | 100.00 (0.00)*^d,e^ |
|  | Overall level | 96.12 (5.03) | 95.30 (8.50) |  | 98.05 (2.99) | 98.48 (3.49) |
| Efficiency (accuracy/RT) | | | | | | |
| ToL | Level 1 | 17.15 (6.39)*^a^ | 20.21 (5.71)*^b,c^ |  | 21.23 (5.37) | 23.62 (4.30)*^d^ |
|  | Level 2 | 12.34 (3.25)*^a^ | 15.08 (5.53)*^c^ |  | 15.57 (3.39) | 16.06 (3.01) |
|  | Level 3 | 6.98 (2.89)*^a^ | 8.45 (3.66)*^b^ |  | 9.56 (3.06) | 11.20 (2.74) |
|  | Level 4 | 6.55 (3.62)*^a^ | 7.54 (2.78) |  | 8.70 (2.20) | 8.91 (2.27) |
|  | Overall level | 9.42 (3.34)*^a^ | 10.86 (3.31)*^b,c^ |  | 12.01 (2.37) | 13.02 (2.11)*^d^ |
| CC1 | Level 1 | 22.48 (3.22) | 21.78 (4.20) |  | 21.68 (2.54) | 22.79 (3.46) |
|  | Level 2 | 14.44 (2.17) | 14.36 (1.59) |  | 14.08 (1.67) | 13.47 (2.31) |
|  | Level 3 | 9.78 (2.52) | 10.15 (1.27) |  | 10.46 (1.19) | 10.16 (1.69) |
|  | Level 4 | 7.59 (1.63) | 7.78 (1.16) |  | 7.48 (1.43) | 7.83 (1.50)*^d^ |
|  | Overall level | 11.74 (1.85) | 11.75 (1.29) |  | 11.69 (1.05) | 11.70 (1.58) |
| CC2 | Level 1 | 28.45 (5.70)*^a^ | 29.03 (5.89) |  | 31.10 (2.68) | 30.81 (3.11) |
|  | Level 2 | 21.10 (3.64)*^a^ | 21.80 (2.64)*^b^ |  | 23.01 (2.27) | 23.02 (1.63) |
|  | Level 3 | 14.51 (2.62)*^a^ | 14.07 (2.96) |  | 15.35 (1.16) | 15.23 (1.06) |
|  | Level 4 | 12.83 (1.84) | 12.53 (1.25) |  | 12.79 (0.99) | 13.15 (0.42) |
|  | Overall level | 17.35 (2.72)*^a^ | 17.28 (2.36)*^b^ |  | 18.29 (1.05) | 18.34 (0.93) |

Response time (RT) and accuracy at the overall level were estimated by collapsing/averaging across all four levels of load for each task condition. The efficiency score was estimated by dividing accuracy by RT; higher scores indicate higher performance efficiency. The Mann-Whitney U test and the Wilcoxon test were used to examine differences between the two groups and differences between the two scan times, respectively. Data are shown as the mean and standard deviation (SD). Asterisks (*) indicate significant differences at p < 0.05: ^a^ patients versus HCs at baseline; ^b^ patients versus HCs at follow-up; ^c^ baseline versus follow-up in patients; ^d^ baseline versus follow-up in HCs; ^e^ differences in patients versus HCs (follow-up minus baseline).

**Supplementary Figures**


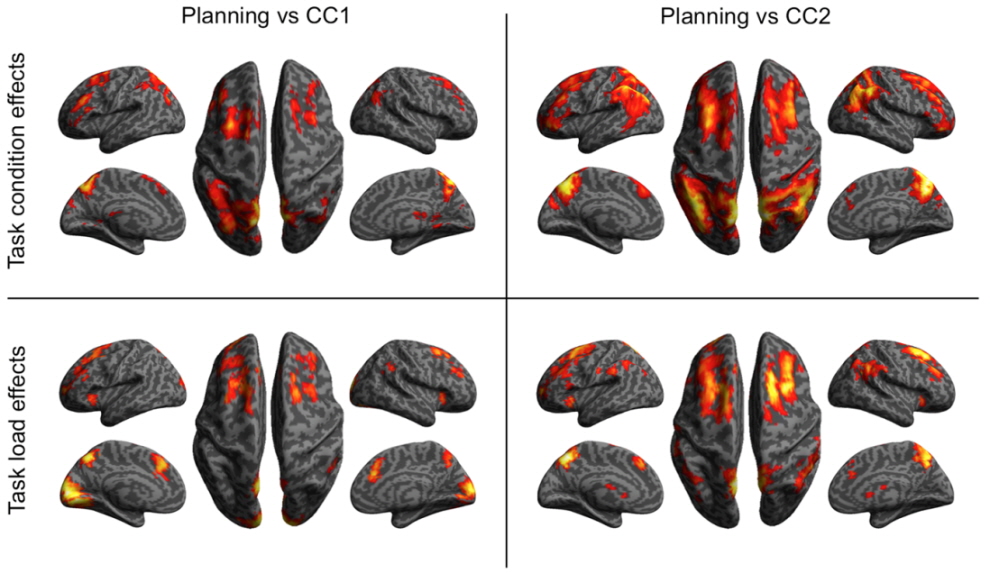


**Supplementary Figure S1.** Activation maps for the main effects of task and load across groups using statistical parametric mapping (SPM) 12 software. Planning was related to increased activation in the dorsolateral prefrontal cortex, premotor cortex, striatum, thalamus, and visuospatial areas (precuneus, superior/inferior parietal and superior occipital cortices) across groups. Increasing task load was correlated with increased activation in the dorsolateral prefrontal cortex, premotor, precuneus, striatum, superior/inferior parietal cortex, anterior insula and cingulate cortices, and visual areas.


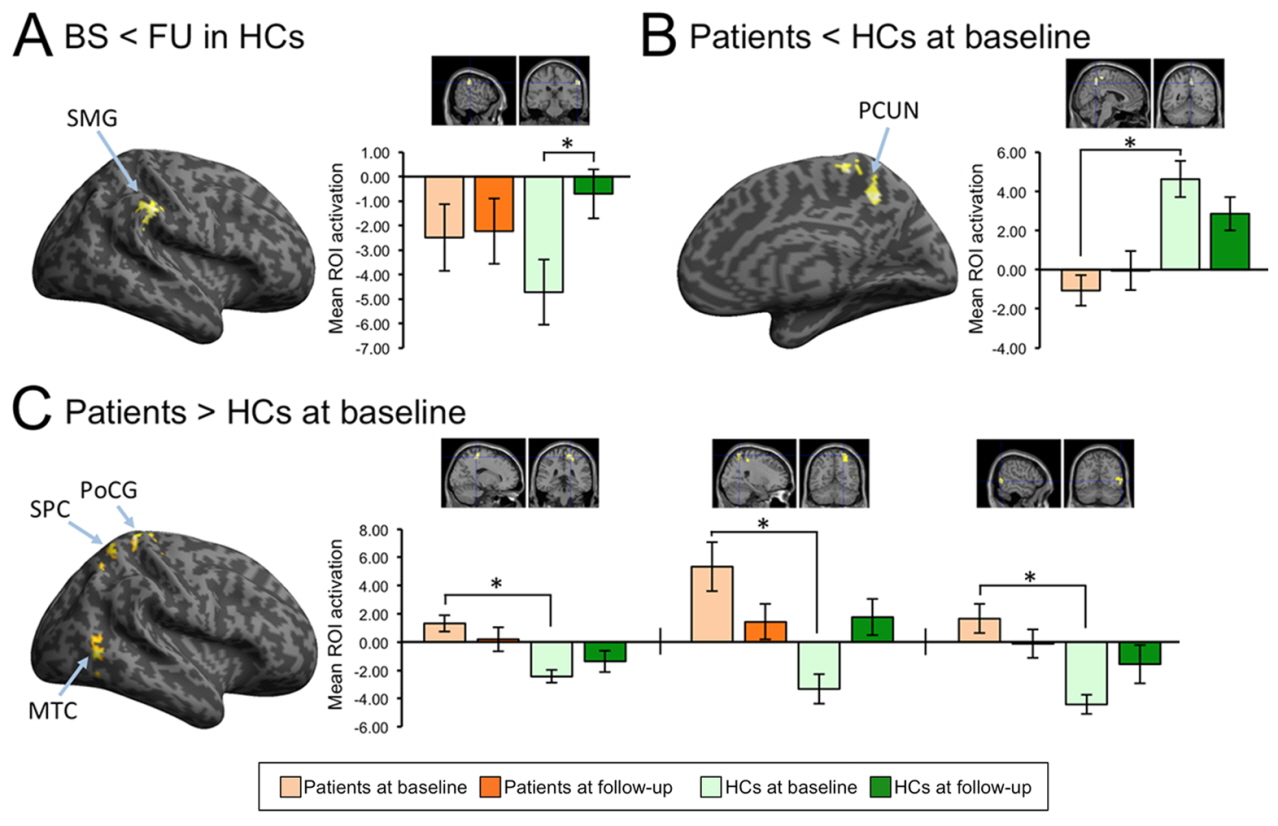


**Supplementary Figure S2**. Results from the univariate GLM analysis at the whole-brain voxel level using statistical parametric mapping (SPM) 12 software. (**A**) A significant time difference in healthy controls (HCs). HCs showed less activation at baseline than at follow-up in the supramarginal gyrus (SMG) for the contrast of the ToL task versus CC1 task across all difficulty levels. (**B** and **C**) Significant differences between the two groups. (**B**) The patients at baseline showed less activation than the HCs in the precuneus/paracentral lobule (PCUN) for the contrast of the ToL task versus CC2 task across all difficulty levels. (**C**) The patients at baseline showed greater activation than the HCs at baseline in the postcentral gyrus (PoCG), superior parietal cortex (SPC), and middle temporal cortex (MTC) for the contrast of the ToL load versus CC2 load, that is, with increasing task load.


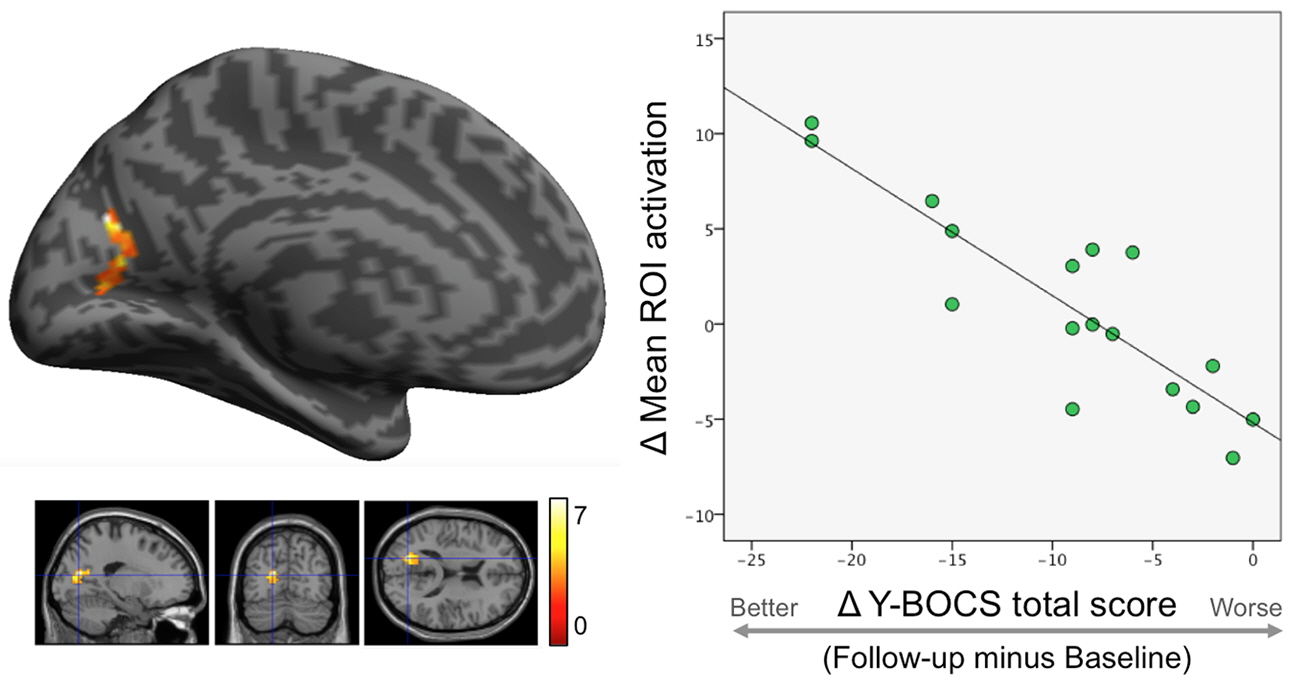


**Supplementary Figure S3**. A significant correlation between changes in clinical symptoms and in neural activity at the whole-brain voxel level. Decreases in patient symptom severity (Y- BOCS total score) were associated with increased activation in the left calcarine cortex (x, y, z = - 18, -72, 15; t-/z-value = 7.88/4.88) for the contrast of the planning task versus control condition 1 using statistical parametric mapping (SPM) 12 software. A scatter plot is shown for illustration purposes only.
